# Clinical significance of mitogen-activated protein kinase kinase kinases in hepatitis B virus -related hepatocellular carcinoma and underlying mechanism exploration

**DOI:** 10.1080/21655979.2022.2037224

**Published:** 2022-03-21

**Authors:** Zhengqian Liu, Xin Zhou, Peng Zheng, Chenheng Bu, Xiao’ou Yan, Haizhou Yu, Yong Xu

**Affiliations:** a Yancheng First Hospital, Affiliated Hospital of Nanjing University Medical School; The First people’s Hospital of Yancheng; b Department of Burn and Plastic Surgery, The First people’s Hospital of Yancheng, Yancheng, P. R. China; c Department of Hepatobiliary Surgery, The First Affiliated Hospital of Guangxi Medical University, Nanning, P. R. China

**Keywords:** Clinical significance, MAP3Ks, HCC

## Abstract

The purpose of this research was to explore the diagnostic/prognostic significance and prospective molecular mechanisms of mitogen-activated protein kinase kinase kinases (*MAP3Ks*) in hepatitis B virus (HBV)-related hepatocellular carcinoma (HCC). Diagnostic/prognostic significance of *MAP3Ks* was screened in the GSE1450 data set and validated in the Guangxi cohort. Various bioinformatics tools were used to explore the biological functions of prognosis-related genes. Subsequently, molecular biology assays were used to verify the biological functions and molecular mechanisms of specific gene. *MAP3K9* was observed to be differentially expressed in HCC and adjacent tissues with satisfactory diagnostic value. It was discovered in survival analysis that *MAP3K13* and *MAP3K15* were associated with overall survival (OS) of patients with HBV-related HCC in the GSE1450 data set and the Guangxi cohort. Nomograms were established based on prognosis-related genes and clinical factors for individualized risk assessment. The assays on HCC cells demonstrated that MAP3K13 regulated the death and proliferation of HCC cells by activating the JNK pathway and inducing the expression of apoptosis-related factors. In conclusion, our results suggested that *MAP3K9* might serve as a diagnostic biomarker in HBV-related HCC and *MAP3K13* and *MAP3K15* might serve as useful prognostic biomarkers. Besides, cytological assays prompted that MAP3K13 might impact the prognosis of HCC by regulating the JNK pathway and inducing apoptosis.

## Graphical abstract

**Figure:**
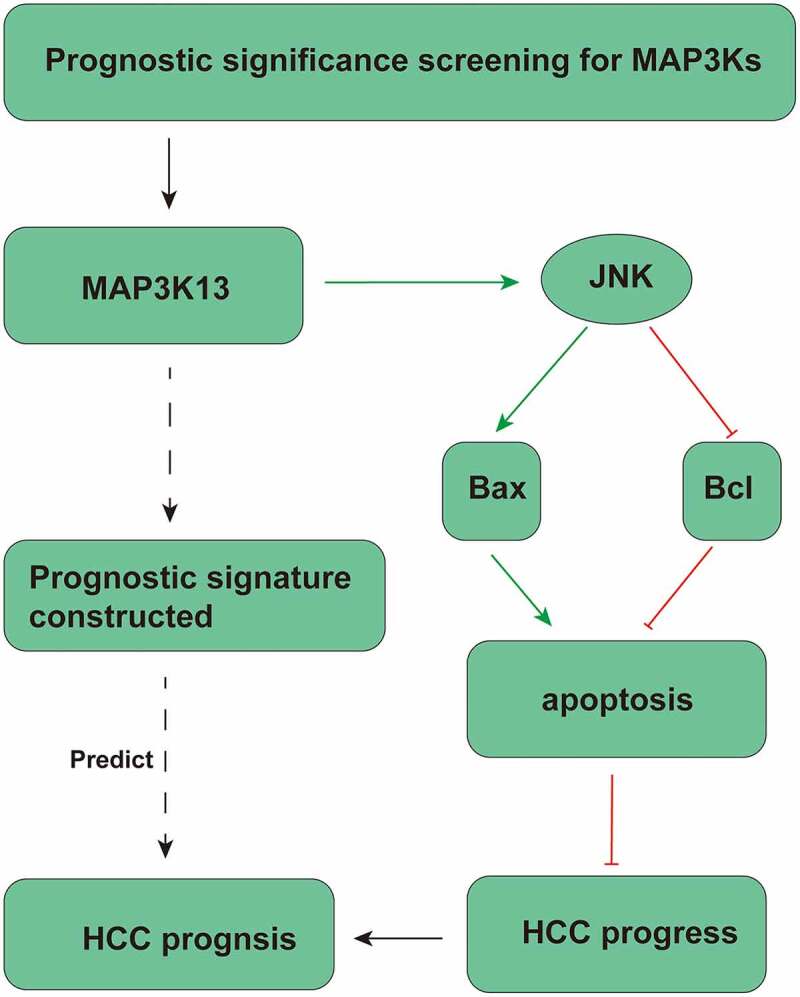

## Introduction

Globally, liver cancer has been reported as the second leading contributor to cancer-related death and its morbidity ranks fourth among all cancers [1]. The inconsistent ranking between mortality and morbidity suggests that there is relatively bad prognosis for patients afflicted with hepatocellular carcinoma (HCC); however, the relatively high ranks for both mortality and morbidity clearly have consequential effects and socioeconomic burdens, and depending upon measures, indexes, and the types of quantitative terms used, the ranks for mortality and morbidity can nearly become equal to one another [1,2]. Some of the highest ranks for HCC have been identified in populations from the Asian-Pacific region, especially eastern Asian countries [3]. Perhaps in line with such findings, in another study, China was found to have accounted for almost half of all the global cases of HCC [4]. Hepatitis B virus (HBV), hepatitis C virus (HCV), aflatoxin, and abuse of alcohol were found to become the leading causes related to the incidence of liver cancer for patients from China [5]. Among these patients, rates of death for liver cancer related to chronic HBV infection have reached 65.9% in male and 58.4% in female [5]. Thus, the researchers reported that there were complex underlying functions of HBV that they acted as key components in tumorigenesis, which might have resulted from circuitous types of mechanisms. HBV nucleotide sequence integration into the host genome was also found to have led to consequent genome instability and mutations in the host cells that directly induced the expression of several cancer-related genes [6,7]. The overexpression of HBx and variations of the related types of preS/S envelope proteins could facilitate dysregulation of transcription and cell proliferation, whereby one result could be that liver cells become sensitive to oncogenically related factors [8]. Accounts of silencing of p53 have been commonly accepted for assessments of tumors associated with HBV infection [9,10]. Reports have also indicated that there was significant ascension in rates of chromosomal alteration based upon examinations of HCC patients afflicted by HBV-related tumors ad patients who were not [6,11]. Compared with non-HBV-infected patients, chronic HBV-afflicted patients are 10–25 times more likely to subsequently develop liver cancer [12]. Furthermore, patients with chronic HBV infections tend to develop more remarkably progressed cases of HCC at relatively younger ages than HCC cases that were solely related to NAFLD and alcohol abuse [12,13].

**Table 1.** Prognostic values of MAP3K gene expression for RFS in HBV-related HCC of GSE14520

		RFS					
Gene expression	Patients (n = 212)	No.. of events	MRT (months)	Crude HR (95% CI)	Crude P	Adjusted HR (95% CI) §	Adjusted P †
MAP3K1							
Low	106	52	53	1		1	
High	106	64	31	1.271(0.881–1.834)	0.198	1.137(0.783–1.650)	0.500
MAP3K2							
Low	106	53	55	1		1	
High	106	63	44	1.101(0.764–1.587)	0.605	1.029(0.714–1.485)	0.877
MAP3K3							
Low	106	58	48	1		1	
High	106	58	42	1.088(0.7561.565)	0.651	1.010(0.698–1.460)	0.959
MAP3K4							
Low	106	65	38	1		1	
High	106	51	55	0.757(0.525–1.093)	0.136	0.761(0.522–1.109)	0.155
MAP3K5							
Low	106	65	33	1		1	
High	106	51	55	0.702(0.487–1.014)	0.058	0.730(0.504–1.056)	0.094
MAP3K6							
Low	106	57	50	1		1	
High	106	59	36	1.094(0.756–1.584)	0.328	1.072(0.741–1.552)	0.712
MAP3K7							
Low	106	66	31	1		1	
High	106	50	58	0.688(0.476–0.995)	0.045	0.901(0.612–1.326)	0.596
MAP3K8							
Low	106	55	48	1		1	
High	106	61	36	1.210(0.840–1.742)	0.305	1.192(0.821–1.730)	0.356
MAP3K9							
Low	106	62	41	1		1	
High	106	54	50	0.930(0.646–1.340)	0.697	0.867(0.602–1.274)	0.498
MAP3K10							
Low	106	59	38	1		1	
High	106	57	52	0.860(0.597–1.237)	0.415	0.860(0.596–1.241)	0.420
MAP3K11							
Low	106	58	47	1		1	
High	106	58	41	1.086(0.755–1.563)	0.656	0.939(0.644–1.368)	0.743
MAP3K12							
Low	106	55	46	1		1	
High	106	61	47	1.102(0.765–1.587)	0.602	1.387(0.949–2.028)	0.091
MAP3K13							
Low	106	64	29	1		1	
High	106	52	55	0.680(0.472–0.982)	0.038	0.735(0.507–1.066)	0.105
MAP3K14							
Low	106	59	36	1		1	
High	106	57	50	0.866(0.601–1.246)	0.437	0.861(0.596–1.243)	0.424
MAP3K15							
Low	106	68	30	1		1	
High	106	48	NA	0.529(0.365–0.767)	0.001	0.576(0.396–0.838)	0.004

RFS † Adjusted for gender, cirrhosis, BCLC stage.

**Abbreviation**: MAP3K mitogen-activated protein kinase kinase kinase 1; HBV, hepatitis B virus; HCC, hepatocellular carcinoma; MRT, median recurrence time; RFS, recurrence free survival; HR, hazard ratio; CI, confidence interval; NA, not available.

**Table 2.** Prognostic values of MAP3K gene expression for OS in HBV-related HCC of GSE14520

		OS					
Gene expression	Patients (n = 212)	No.. of events	MST (months)	Crude HR (95% CI)	Crude P	Adjusted HR (95% CI)	Adjusted P ‡
MAP3K1							
Low	106	33	NA	1		1	
High	106	49	60.5	1.443(0.928–2.244)	0.104	1.315(0.837–2.267)	0.235
MAP3K2							
Low	106	35	NA	1		1	
High	106	47	NA	1.258(0.812–1.949)	0.304	1.222(0.781–1.913)	0.380
MAP3K3							
Low	106	43	NA	1		1	
High	106	39	NA	0.908(0.588–1.401)	0.662	0.785(0.502–1.227)	0.289
MAP3K4							
Low	106	44	NA	1		1	
High	106	38	NA	0.907(0.588–1.400)	0.659	0.848(0.542–1.328)	0.472
MAP3K5							
Low	106	49	60.5	1		1	
High	106	33	NA	0.609(0.392–0.947)	0.028	0.664(0.418–1.053)	0.081
MAP3K6							
Low	106	39	NA	1		1	
High	106	43	NA	1.174(0.757–1.819)	0.474	1.256(0.811–1.946)	0.308
MAP3K7							
Low	106	46	NA	1		1	
High	106	36	NA	0.762(0.492–1.179)	0.222	1.053(0.660–1.681)	0.829
MAP3K8							
Low	106	37	NA	1		1	
High	106	45	NA	1.286(0.832–1.986)	0.258	1.120(0.702–1.789)	0.634
MAP3K9							
Low	106	44	NA	1		1	
High	106	38	NA	0.883(0.572–1.362)	0.573	0.781(0.499–1.222)	0.279
MAP3K10							
Low	106	41	NA	1		1	
High	106	41	NA	0.948(0.615–1.463)	0.811	0.928(0.601–1.433)	0.736
MAP3K11							
Low	106	42	NA	1		1	
High	106	40	NA	0.960(0.622–1.480)	0.852	0.680(0.424–1.091)	0.11
MAP3K12							
Low	106	43	NA	1		1	
High	106	39	NA	0.876(0.568–1.353)	0.551	1.181(0.749–1.862)	0.473
MAP3K13							
Low	106	49	57.9	1		1	
High	106	33	NA	0.572(0.368–0.890)	0.013	0.610(0.389–0.958)	0.032
MAP3K14							
Low	106	47	NA	1		1	
High	106	35	NA	0.660(0.426–1.023)	0.063	0.738(0.463–1.177)	0.203
MAP3K15							
Low	106	51	57.9	1		1	
High	106	31	NA	0.482(0.308–0.753)	0.001	0.558(0.354–0.878)	0.012

OS ‡ Adjusted for AFP, cirrhosis, BCLC stage and tumor size.

**Abbreviation**: MAP3K mitogen-activated protein kinase kinase kinase 1; HBV, hepatitis B virus; HCC, hepatocellular carcinoma; MST, median survival time; OS, overall survival; HR, hazard ratio; CI, confidence interval; NA, not available.

One aspect related to the dynamics underlying HBV-related HCC is the MAPK signaling pathway [14]. The MAPK signaling pathway includes a series of cytoplasmic phosphokinases that transduce and transmit signals related to levels of extracellular mitogen availability to the nucleus of the cell. Receptor-linked cytoplasmic tyrosine kinases are activated when a signaling molecule binds to the cell-surface receptor [15]. The MAPK signaling pathway is similarly initiated by epidermal growth factor (EGF), whereupon EGFR becomes activated by EGF, and then Ras becomes activated by swapping its GDP for a GTP. This was followed by the activated form of Ras serving to motivate MAP3K, whereby MAP3K facilitates phosphorylation and activation of MAP2K, MAP2K itself becomes phosphorylated, and the ultimate form is an activated MAPK [15–18]. This MAP3K-MAP2K-MAPK module serves as crucial medium for transducing information contained in extracellular stimuli into certain cellular responses, including such as differentiation, proliferation, and apoptosis [19–22]. As MAP3K family members are indispensable part of the MAPK pathway, we hypothesized that MAP3K family members may play an important role in HCC. There were no researchers who have explored the molecular function and clinical significance of MAP3K family members in HCC so far. This research systematically analyzed the prognostic value of all members of the MAP3K family in HCC and then conducted bioinformatic and molecular biology studies on the regulation mechanisms of prognostically related genes.

## Materials and methods

### Data acquisition

Clinical features of 247 HCC-afflicted patients and whole transcriptome sequencing-based data from these patients in GSE1450 were acquired from the GEO database (accessed: 27 January 2020). As the main purpose of our study was to investigate measures of clinical significance of *MAP3K*s in HBV-related HCC, patients without HBV infection were eliminated.

### Tissue specimen collection

HCC and paracancerous tissues of 54 HBV-related HCC patients were collected in the First Affiliated Hospital of Guangxi Medical University (from 2018–03-22 to 2018–10-01). The tissue samples were immersed in the RNA*later* Solutions immediately after separation (Thermo Fisher Scientific, USA). These tissue specimens were stored in the −80 refrigerator at ordinary times. This investigation had been approved by the ethics committee of Guangxi Medical University, the first affiliated hospital (Approval number: 2015 [KY‐E‐032]). All patients had been informed and had signed the informed consent before surgery.

### Bioinformatic analyses of MAP3Ks

DAVID 6.8 (The Database for Annotation, Visualization and Integrated Discovery, accessed at 28 January 2020) was used for functional-based annotations for *MAP3Ks* [23,24]. In the options for annotations in ‘Summary Results’, we selected the KEGG database and gene ontology database. The function annotation chart was visualized in R Studio (Version 1.2.5033) using R packages: *GOplot* [25], *Hmisc* [26], and *ggplot2*v [27].

### Differential expression analysis and assessment of diagnostic value

GTEx Portal (https://www.gtexportal.org/) was used to acquire the expression of *MAP3Ks* in multiple human tissues [28]. Unpaired t-tests were applied in order to inspect the expression levels of *MAP3Ks* between HCC and paracancerous tissues in GSE14520. We used Receiver Operating Characteristic (ROC) curves to assess measures of diagnostic efficiency of the *MAP3Ks* in GSE14520.

### Survival analysis

The Kaplan-Meier (K-M) method with the log-rank test was applied to perform univariate survival analysis in GSE14520. It was used to analyze the relationship between *MAP3K* family genes and OS (overall survival)/RFS (recurrence-free survival). Besides, the Cox proportional hazards model was used to calibrate the impact of clinical variables for *MAP3Ks* in multivariate survival analysis.

Genes associated with prognostic significance were further integrated in combined effect survival analysis. A new variable was constructed based upon the expression levels of multiple prognosis-related *MAP3K*s, and then the relationship between the variable and prognosis was explored.

### qRT‑PCR

Total RNAs were isolated from HCC tissues and paracancerous tissues using the RNA Isolation solvent (Omega Bio-tek, Georgia, USA) according to the protocol. Isolated RNAs were reverse-transcribed into complementary DNA with the use a PrimeScript^TM^ RT reagent kit (Takara, Dalian, China). Quantitative real-time PCR (qRT-PCR) was performed on the QuantStudio 6 Flex Fluorescence Quantitative PCR Device (Thermo Fisher Scientific, Waltham, MA, USA) using SYBR Green Mix (Roche, Switzerland). The ΔΔCt method was used to calculate the relative gene expression. *GAPDH* was used as the internal control for MAP3Ks expression. Primer sequences are as follows: *GAPDH*, antisense prime (5’-CGCCCAATACGACCAAAT-3’), forward prime (5’-GTCAGCCGCATCTTCTTT-3’); *MAP3K9*, antisense prime (5’- AAAGATGGTCGTGAGTGGGG −3’), forward prime (5’- GTGGAGCTATGGGGTGCTAC-3’); *MAP3K13*, antisense prime (5’-GGGCTCCAAAACCTCTCCCA-3’), forward prime (5’-GATCCCCGACAGAACACTGAAAT-3’); and *MAP3K15*, antisense prime (5’-CGCTCATGTCTACCACAGCA-3’), forward prime (5’-GTATACGTGCGCAGTGAGAG-3’)

### Nomogram and prognostic signature construction for HCC

Based upon the results from survival analysis, variables that were found to have been associated with OS/RFS were then incorporated into nomograms for predicting the 1-, 3-, and 5-year OS/RFS [29]. Internal validation using the Bootstrap self-sampling method was applied to help evaluate the predictive power of the model.

In order to calculate risk scores for each patient, we established a prognostic model, which accounted for the expression level of prognostic genes [29]. The calculation of risk scores was based upon a formula as follows: Risk score = expression of gene_1_ x β_1_ + expression of gene_2_ x β_2_+ … expression of Gene_n_ x β_n_ [30,31], where β_n_ is the regression coefficient derived from the result of multivariate Cox proportional hazards regression analyses for the corresponding gene. In consecutive order, we performed survival analyses for high- and low-risk groups. Furthermore, in order to verify measures of reliability and accuracy for the model, we constructed temporally oriented ROC-based curves using *survivalROC* package in R [32].

### Gene set enrichment analysis (GSEA)

We used GSEA to assess trends in distributions of genes included among a predefined set of genes in the phenotypic correlation sequence in order to determine their contribution to the phenotype. According to the characterization of the median levels of expression of *MAP3K13* in the GSE14520 database, 212 HBV-related HCC patients were separated into a high and a low-*MAP3K13* treatment group based upon the relative levels of expression. According to the median levels of expression of *MAP3K15* in the GSE14520 database, 212 HBV-related HCC patients were separated into high- and low*-MAP3K15* groups based on relative levels of expression. C2-curated gene sets (c2.all.v7.0.symbols.gmt) were selected as the basis factor sets in GSEA_4.0.3 (http://www.broadinstitute.org/gsea). GSEA-derived gene enrichment sets that attained a false discovery rate (FDR) of <0.25 and a *P* value of<0.05 were considered as the level of statistical significance at which enrichment sets with no difference between treatment groups would be rejected.

### Cell transfection

RNA interfering for MAP3K13 was performed by virtue of Si-RNA, which was designed and synthesized by hanbio (https://www.hanbio.net/, Shanghai, China), with the sequence displayed in Table S2. Lipofectamine 3000 Transfection Reagent and Opti-MEM medium were purchased for Si-RNA transfection (Thermo Fisher Scientific, shanghai, China). Transfection was performed following the manufacturer’s protocol. Transfection efficiency was assessed at 48 h.

### Western blot

Western blot assay was performed referring to the protocol of a previous study [33]. Antibodies of GAPDH, JNK, BAX, Bcl, and MAP3K13 were purchased from WUHAN SANYING (Proteintech Group, Inc, USA). Antibody dilution and incubation times were in accordance with the corresponding manufacturer’s protocol.

### CCK-8 assay

After 48 h of transfection, cells were collected for the CCK-8 assay. 2000 cells were added to each well of a 96-well plate, with four more duplicated plates prepared. 100 ul of CCK-8 reagent (DOJINDO, Shanghai, China) was added dropwise into each well. After 2 h of incubation at 37°C in a lucifugal room, the absorbance in 450 nm was detected using a Varioskan LUX microplate reader (Thermo Fisher Scientific, USA). Each assay was repeated three times. Student's t-test was used to examine the statistical significance between the experimental group and the control group.

### Cell colony formation assay

After 48 h of transfection, 300 cells were collected and then added to each well of a 6-well plate. The cells were cultivated with plenty of medium for two weeks in a cell incubator at 37°C with 5% CO_2_. Cells are stained with crystal violet for 20 min. Each assay was repeated three times. Student’s t-test was used to examine the statistical significance between the experimental group and the control group.

### Apoptosis assay

After 48 h of transfection, cells were collected for the apoptosis assay. BH3 hydrochloride (MedChemExpress, Shanghai, China), an apoptosis-inducing polypeptide, which induces apoptosis by activating Bax or neutralizing Bcl-2 [34], was used for apoptosis induction. An AnnexinV-FITC/PI apoptosis kit (BD bioscience, USA) was applied in this study following the manufacturer’s protocol. Each assay was repeated three times. Student’s t-test was used to examine the statistical significance between the experimental group and the control group.

### Statistical analyses

Statistical analyses were all conducted using SPSS version 24.0 (IBM Corporation, Armonk, NY, USA) and R 3.6.2. (https://www.r-project.org/). We used the Kaplan-Meier method with the log-rank test in univariate survival analyses for *MAP3Ks* and clinical features. Cox proportional hazard modeling was used in multivariable survival analyses. With respect to results for the unpaired t tests and log-rank tests and for results from Cox models, *P* values<0.05 were considered statistically significant. FDR control was fulfilled by using the Benjamini–Hochberg procedure and adjusted for multiple testing in GSEA.

## Results

### Data sources

In the GSE14520 data set, 35 patients with nonchronic HBV infection were excluded and 212 HBV-related HCC patients were reserved for subsequent analysis. After eliminating 35 non-HBV-related HCC patients, we obtained a total of 416 tissue samples with integral microarray data and detailed clinical/prognostic data. The 416 samples included 212 samples of tumor-afflicted tissues from HBV-related HCC patients and 204 samples of paracancerous tissues of these 212 patients, with paracancerous tissues of 8 patients absent.

### *Bioinformatic analysis of* MAP3Ks

**Figure 1.**
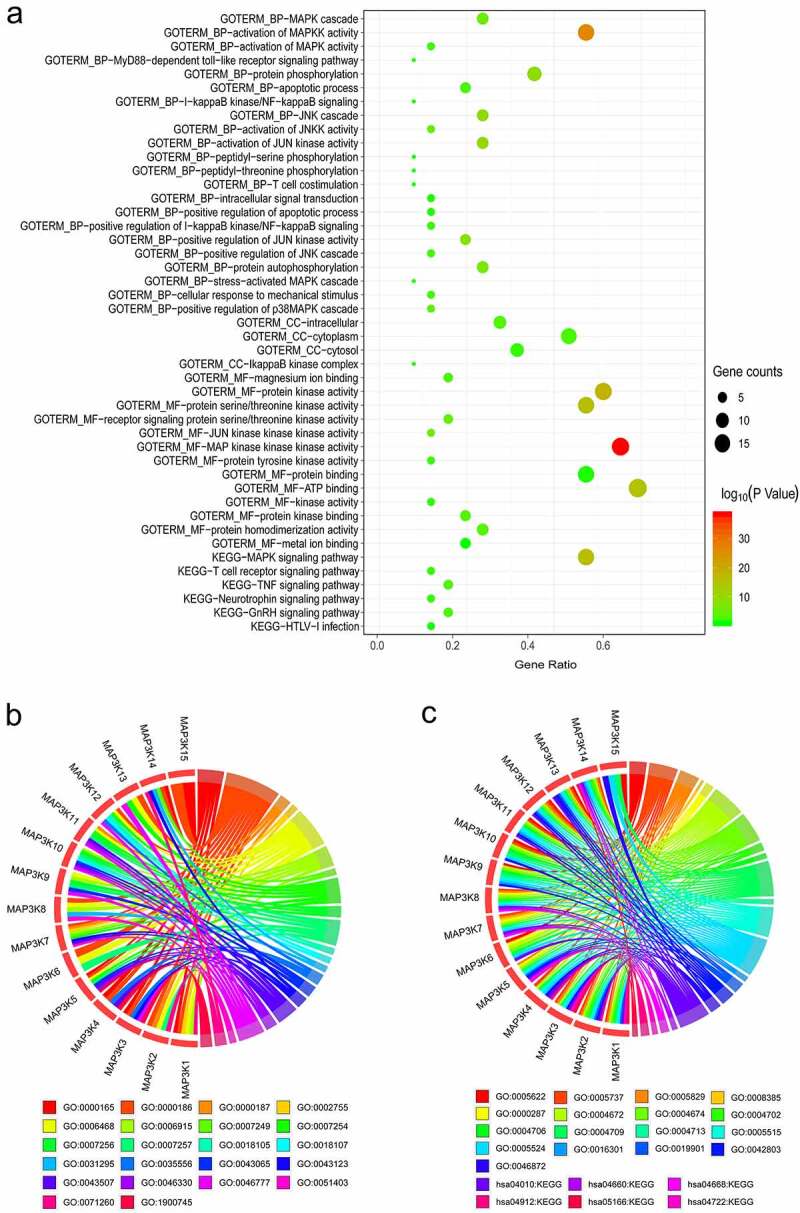
Bioinformatics-based results from DAVID: (a) the pathways, molecular functions, biological processes, and cellular components in which *MAP3Ks* are enriched; (b and c) details of *MAP3Ks* corresponding to specific pathways, molecular functions, biological processes, and cellular components. DAVID, The Database for Annotation, Visualization and Integrated Discovery; *MAP3Ks*, mitogen-activated protein kinase kinase kinases.

We visualized the results of bioinformatic analyses on DAVID’s website. The pathways, molecular functions, biological processes, and cellular components in which *MAP3Ks* are enriched, along with corresponding gene counts and *P* values, are shown in a bubble chart (Figure 1(a)). The enrichment analysis results showed that MAP3K family genes were involved in many important biological pathways, such as MAPK signaling pathway and JUK signaling pathway. Details of *MAP3Ks* corresponding to specific pathways, molecular functions, biological processes, and cellular components are shown in chord diagrams (Figure 1(b,c)).

### Differential expression analysis and assessment of diagnostic value

**Figure 2.**
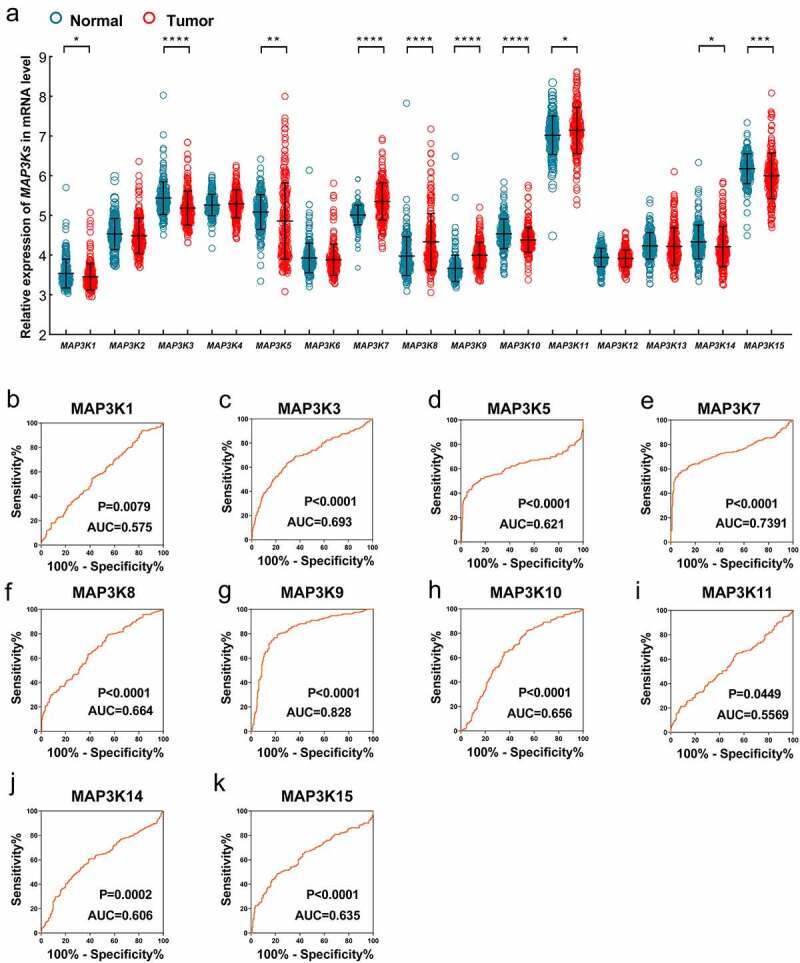
Expression of *MAP3Ks* in HCC and normal live tissues: (a) expression level of *MAP3K1-15* in HCC and normal live tissues; (b) ROC of *MAP3K1*, (c) ROC of *MAP3K3*, (d) ROC of *MAP3K5*, (e) ROC of *MAP3K7*, (f) ROC of *MAP3K8*, (g) ROC of *MAP3K9*, (h) ROC of *MAP3K10*, (i) ROC of *MAP3K11*, (j) ROC of *MAP3K14*, and (k) ROC of *MAP3K15MAP3Ks*, mitogen-activated protein kinase kinase kinases; HCC, hepatocellular carcinoma; ROC, receiver operating characteristic curve.

Differential expression analysis in GSE14520 indicated that expression of *MAP3K1, MAPK3K3, MAP3K5, MAP3K7, MAP3K8, MAP3K9, MAP3K10, MAP3P11, MAP3K14*, and *MAP3K15* was significantly different between HCC and normal liver tissues (Figure 2(a)). Compared with normal liver tissues, *MAP3K1, MAP3K3, MAP3K5, MAP3K10, MAP3K14*, and *MAP3K15* were downregulated in HCC tissues, while *MAP3K7, MAP3K8, MAP3K9*, and *MAP3K11* were upregulated in HCC tissues. Besides, ROC analysis showed that *MAP3K9* gene had satisfactory diagnostic performance in HCC (AUC = 0.829, *P* < 0.001) (Figure 2(g)), and the diagnostic value of other MAP3K in liver cancer was not significant (Figure 2(b–f,h–k)).

### Survival analysis in GSE14520

Gender (*P* = 0.021), cirrhosis (*P* = 0.036), and BCLC stage (*P* < 0.001) were associated with the RFS of HBV-related HCC, and the AFP level (*P* = 0.049), BCLC stage (*P < *0.001), cirrhosis (*P* = 0.041), and tumor size (*P* = 0.002) were independent predictors for OS (Table S1).

**Figure 3.**
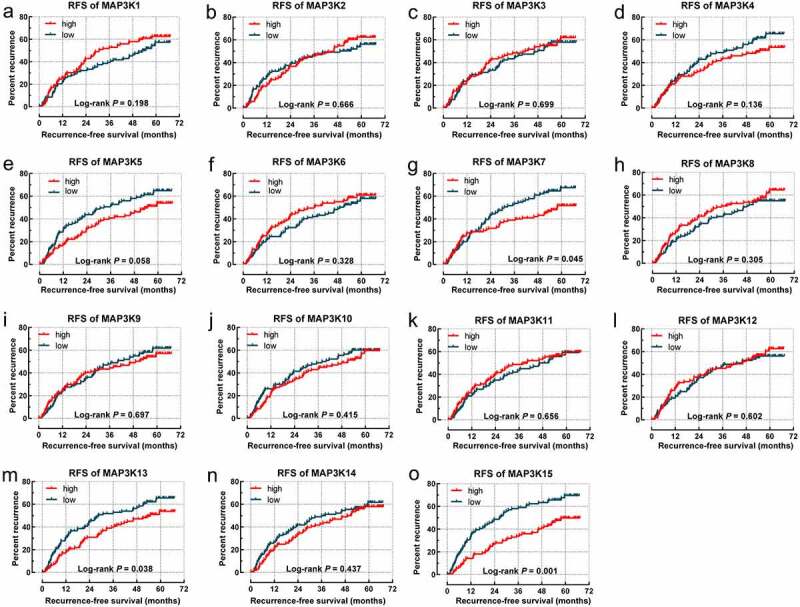
Survival analysis-based results of *MAP3Ks* for RFS in HBV-related HCC: (a–o) Survival curve for *MAP3K1-15* for RFS of HBV-related HCC; *MAP3Ks*, mitogen-activated protein kinase kinase kinases; RFS, recurrence-free survival; HCC, hepatocellular carcinoma.

**Figure 4.**
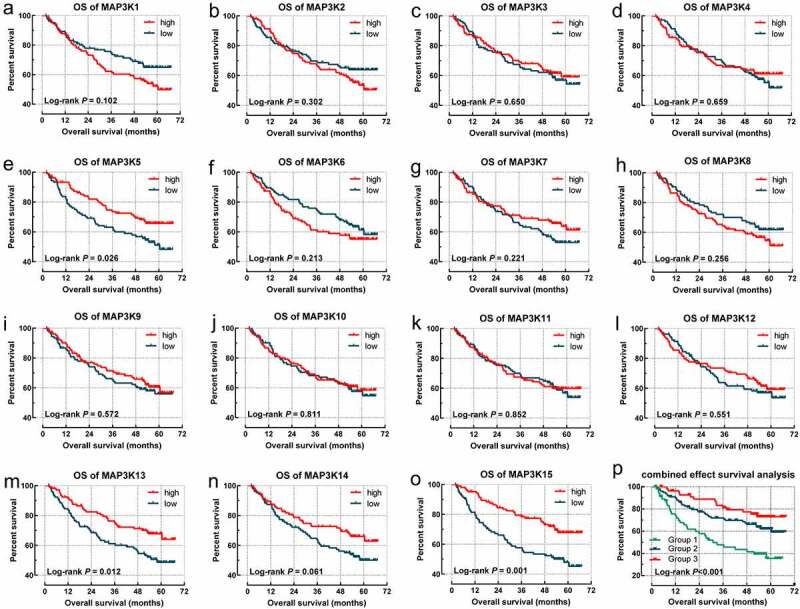
Survival analysis-based results of *MAP3Ks* for OS in HBV-related HCC: (a–o) sSurvival curve for *MAP3K1-15* for OS of HBV-related HCC; (p) joint effect survival analyses of *MAP3K13* and *MAP3K15* for OS of HBV-related HCC. *MAP3Ks*, mitogen-activated protein kinase kinase kinases; OS, Overall survival; HBV, Hepatitis B Virus; HCC, hepatocellular carcinoma.

*MAPK15* (log-rank *P* = 0.001; adjusted *P* = 0.004; Table 1, Figure 3(o)) was associated with RFS of HBV-related HCC. *MAP3K13* (log-rank *P* = 0.013; adjusted *P* = 0.032; Table 2, Figure 4(m)) and *MAPK15* (log-rank *P* = 0.001; adjusted *P* = 0.012; Table 2, Figure 4(o)) were associated with OS of the HBV-related HCC.

We integrated *MAP3K13* and *MAP3K15* into joint survival analysis. With high levels of expression of both *MAP3K13* and *MAP3K15* observed to be associated with better outcomes in OS, we preliminarily considered that both genes played a beneficial role in HBV-related HCC. Patients were divided into three groups (group IDs: 1, 2, and 3) based upon the grouping methods further outlined in Table 3. There were significant differences among the OS of groups 1, 2, and 3 (*P* < 0.001; Table 3 and Figure 4(p)). The HCC patients in group 1 with high expression of *MAP3K13* and *MAP3K15* tend to be accompanied by the worst outcome.

**Table 3.** Joint effects analysis of MAP3K13 and MAP3K15 expression for OS in in HBV-related HCC of GSE14520

Group	MAP3K13	MAP3K15	Patients	No. of events	MST (Months)	Crude HR (95% CI)	Crude P	Adjusted HR (95% CI)	Adjusted P ¶
1	Low	Low	56	32	31	1		1	
2	Low	High	100	36	NA	0.473(0.294-0.763)		0.606(0.366-1.003)	
	High	Low							
3	High	High	56	14	NA	0.295(0.157-0.554)	<0.001	0.369(0.195-0.700)	0.002

OS ¶ Adjusted for tumor size, cirrhosis, BCLC stage, and AFP in GSE14520. **Abbreviation**: MAP3K, mitogen-activated protein kinase kinase kinase; HCC, hepatocellular carcinoma; OS, overall survival; MST, median survival time; HR, hazard ratio; CI, confidence interval; NA, not available.

### Nomogram and prognostic signature

**Figure 5.**
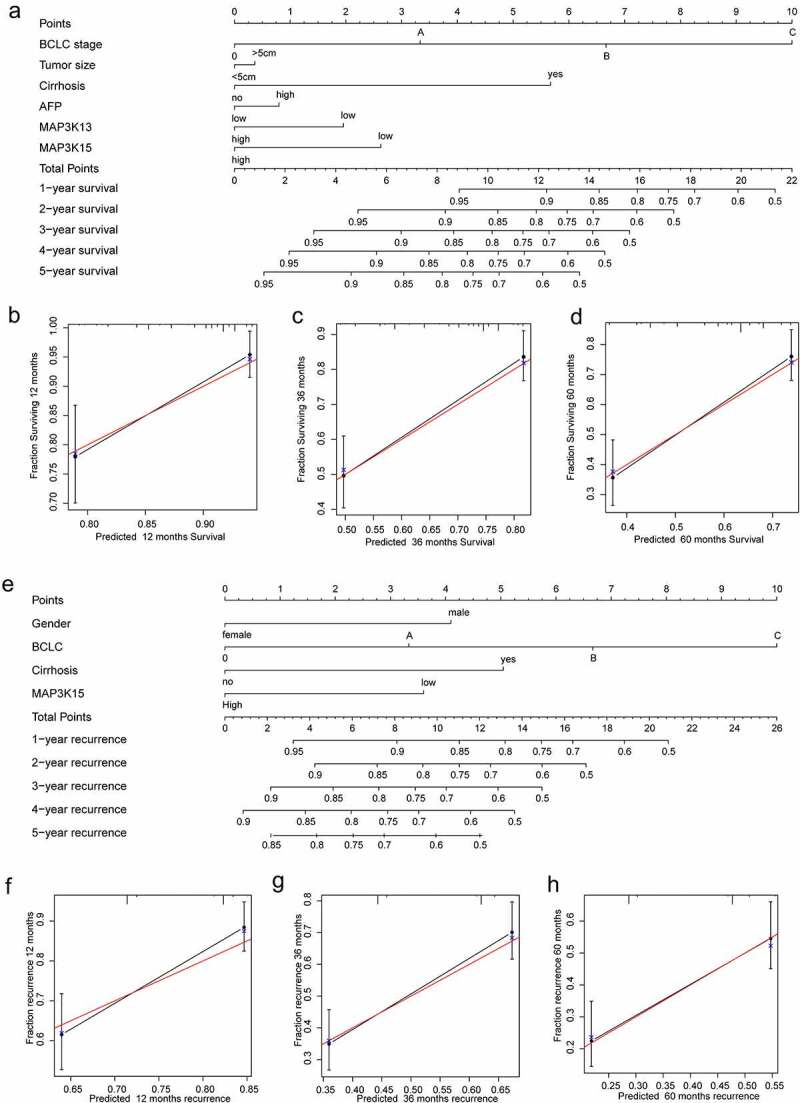
Nomogram for predicting 1-, 2-, and 3-year OS/RFS of HCC: (a) Nomogram for OS; (b–d) verification model for Nomogram in 1-, 2-, and 3-year OS, respectively. and (e) Nomogram for RFS; (f–h) verification model for Nomogram in 1-, 2-, and 3-year RFS, respectively. OS, overall survival; RFS, rrecurrence-free survival; HCC, hepatocellular carcinoma.

The levels of AFP, the stage of BCLC, status of Cirrhosis, tumor size, levels of *MAP3K13*, and levels of *MAP3K15* were incorporated into a nomogram for a visual representation of OS (Figure 5(a)). The curves from the prediction group and the observation-based group fit well with respect to one another and OS rates (Figure 5(b–d)).

Gender, the stage of BCLC, status of cirrhosis, and *MAP3K15* were incorporated into the nomogram for RFS (Figure 5(e)). The curves of the prediction group and the observation-based group fit well with respect to one another and RFS rates (Figure 5(f–h)).

**Figure 6.**
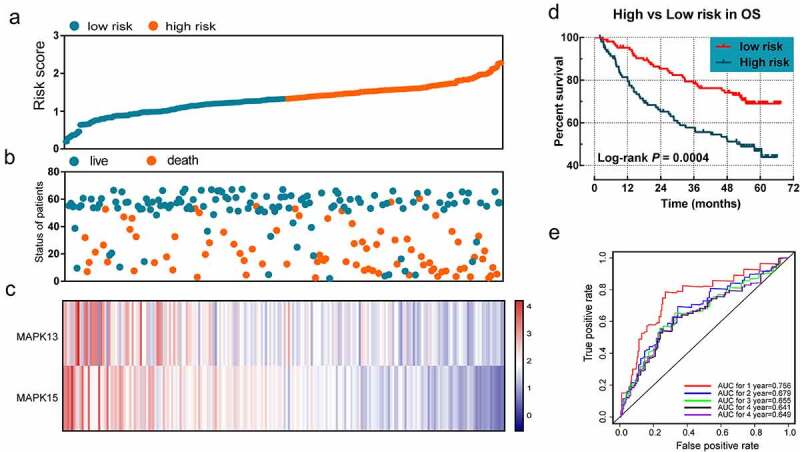
The prognostic signature in terms of expression of *MAP3K13* and *MAP3K15* for HBV-related HCC: (a) risk score plot; (b) survival status scatter plot; (c) heat map of the levels of expression of *MAP3K13* and *MAP3K15* in low- and high-risk groups; (d) Kaplan-Meier curves for low- and high-risk groups; and (e) receiver operating characteristic curve for predicting 1-, 2-, and 3-year survival rates in patients with HBV-related HCC by risk score. *MAP3K*, mitogen-activated protein kinase kinase kinase. HBV, Hepatitis B Virus; HCC, hepatocellular carcinoma.

*MAP3K13* and *MAP3K15* were selected for evaluating the prognostic signature of OS. The regression coefficients for *MAP3K13* and for *MAP3K15* were −0.488 and −0.550, respectively. The formula used for the risk in OS was as follows: risk score = expression value of *MAP3K13* × −0.488 + expression value of *MAP3K15* × −0.550. Survival analyses indicated that the risk score was significantly related to the OS rate (log-rank *P < *0.001). The prognostic signature effectively predicted the OS rate of HBV-related HCC patients (1-year AUC = 0.756, 2-year AUC = 0.679, 3-year AUC = 0.655, 4-year AUC = 0.641, and 5-year AUC = 0.649; Figure 6).

### GSEA

**Figure 7.**
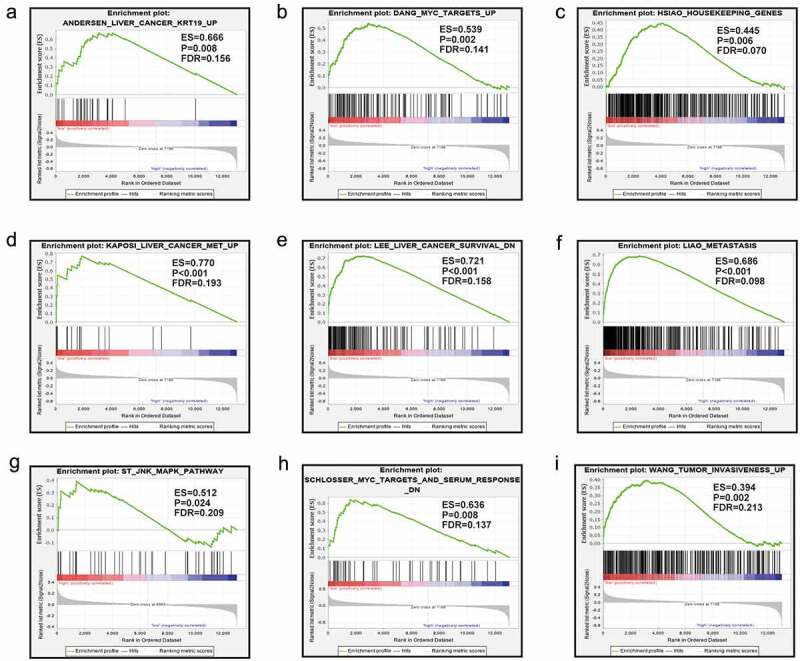
GSEA in terms of *MAP3K13* in GSE14520 based on C2-curated gene sets. GSEA, Gene Set Enrichment Analysis; C2 curated gene sets, the C2 collection contains two subcollections: chemical and genetic perturbations (CGPs) and canonical pathways (CPs).

**Figure 8.**
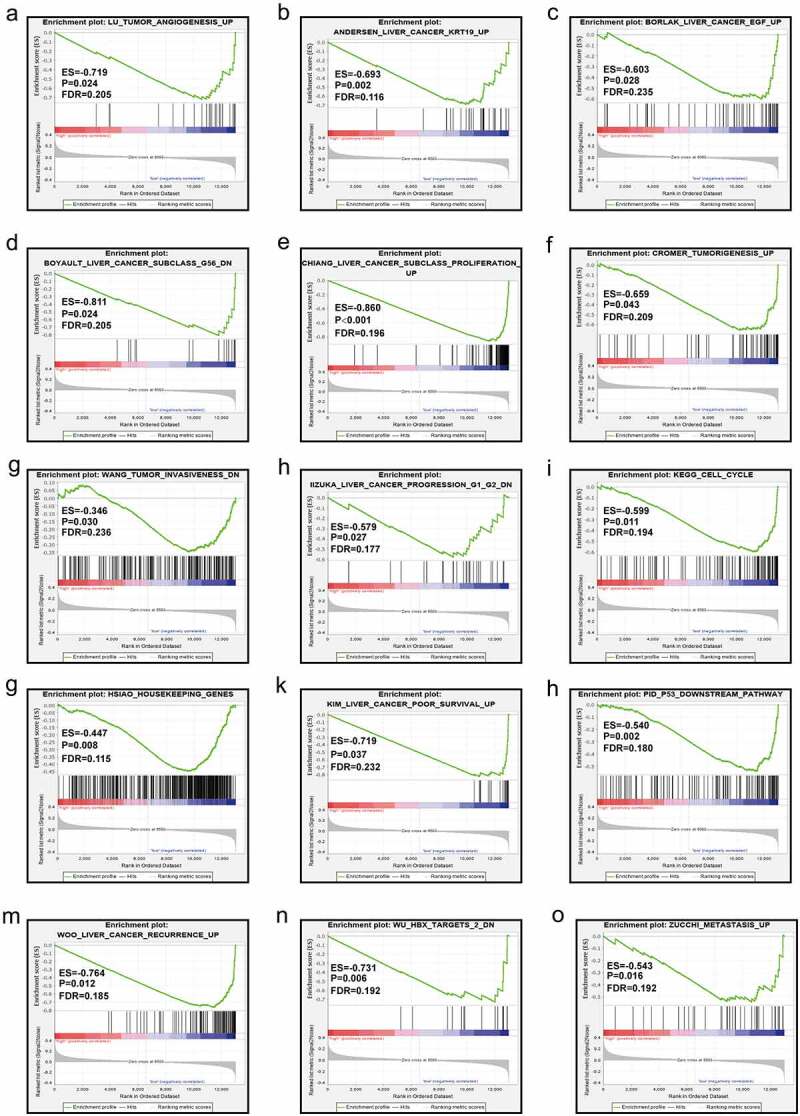
GSEA in terms of *MAP3K15* in GSE14520 based on C2-curated gene sets. GSEA, Gene Set Enrichment Analysis; C2-curated gene sets, the C2 collection contains two subcollections: chemical and genetic perturbations (CGPs) and canonical pathways (CPs).

The results from GSEA indicated that the low expression level of *MAP3K13* was involved in the dynamics underlying liver cancer including the myc pathway, JNK pathway, and metastasis, resulting in the decrease in liver cancer survival rates. (Figure 7). Furthermore, *MAP3K15* was associated with liver cancer subclasses, tumorigenesis, cell cycle dynamics, tumor angiogenesis, the progression of liver cancer, and increased rates of recurrence of liver cancer (Figure 8).

### Validation in the Guangxi cohort

**Figure 9.**
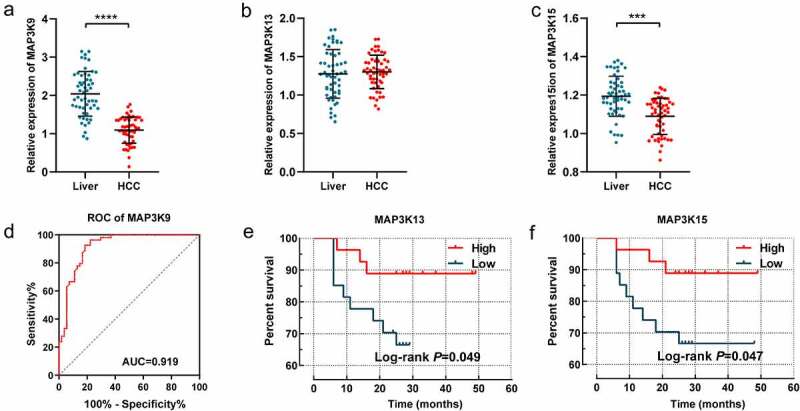
Validation of the diagnostic/prognostic significance of *MAP3Ks* in the Guangxi cohort: (a-c) relative expression of *MAP3K9, MAP3K13,* and *MAP3K15* between HCC and normal liver tissues; (d) ROC of *MAP3K9*; (e) survival curve of *MAP3K13*; and (E) survival curve of *MAP3K15.*

The expression difference of *MAP3K9, MAP3K13*, and *MAP3K15* between HCC tissues and adjacent normal tissues was further assessed in the Guangxi cohort. Besides, prognostic significance of *MAP3K13* and *MAP3K15* was also further demonstrated in 54 patients hospitalized in the First Affiliated Hospital of Guangxi Medical University. It is consistent with the result of GSE14520 that *MAP3K9* was observably more highly expressed in HCC tissues, compared with paracarcinoma tissues (*P* < 0.001, Figure 9(a)); The diagnostic performance of *MAP3K9* was also superior in the Guangxi cohort (*P* < 0.001, AUC = 0.696, Figure 9(d)). There was no significant expression difference of *MAP3K13* observed between HCC tissue and normal liver tissues. High expression of *MAP3K13* (*P* = 0.049, Figure 9(e)) and *MAP3K15* (*P* = 0.049, Figure 9(f)) was respectively demonstrated to be associated with better prognosis of HCC patients in the Guangxi cohort.

### Biological function of MAP3K13 in HCC cell lines

**Figure 10.**
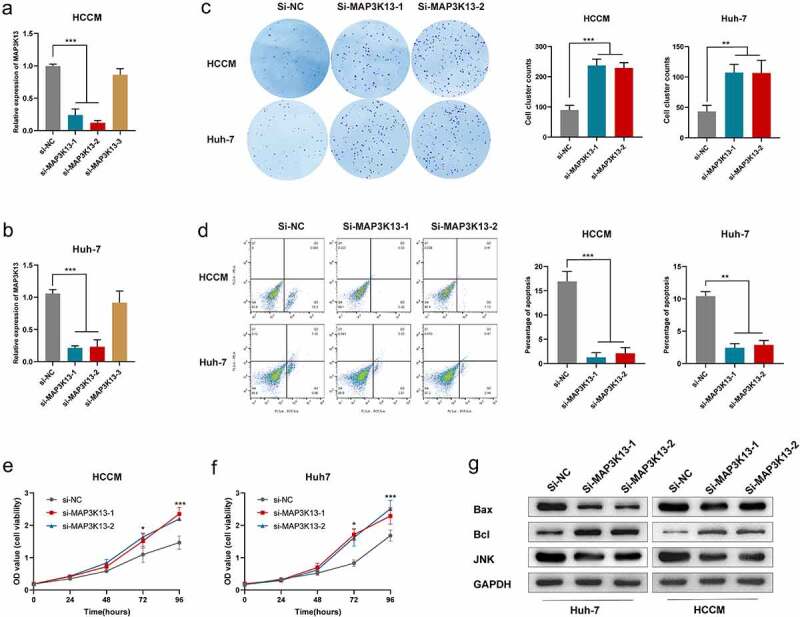
Effects of *MAP3K13* knockdown on HCC cells: xpression level of *MAP3K13* in HCCM cells (a) and Huh-7 cells (b) after si-MAP3K13/si-NC transfection; (c) the number of colonies of HCCM and Huh-7 cells after two weeks of transfection; (d) under intervention of BH3 hydrochloride (5uM), the apoptosis ratio of HCCM cells and Huh-7 cells after si-MAP3K13/si-NC transfection; cell viability curve of HCCM cells (e) and Huh-7 cells (f) after si-MAP3K13/si-NC transfection; (g) expression level of JNK, Bcl, and Bax in HCCM cells and Huh-7 cells after si-MAP3K13/si-NC transfection. * P < 0.05, ** P < 0.01, *** P < 0.001, and **** P < 0.0001.

The survival analysis results suggested that MAP3K13 was a protective factor in HCC, and the bioinformatic analysis revealed that MAP3K13 was associated with the JNK signal pathway (Figures 1(a) and 7(g)). Three si-RNAs were designed by the manufacturer for *MAP3K13*, among which the knockdown efficiencies of si-1 and si-2 were satisfactory in HCCM cells and Huh-7 cells (Figure 10(a,b)). The colony formation ability of HCCM cells and Huh-7 cells was significantly enhanced after si-MAP3K13 transfection (Figure 10(c)). The apoptosis of HCCM cells and Huh-7 cells was significantly restrained after si-MAP3K13 transfection under intervention of BH3 hydrochloride (5uM) (Figure 10(d)). The proliferation of HCCM cells and Huh-7 cells was significantly promoted after si-MAP3K13 transfection (Figure 10(e,f)). Thus, it could be seen that MAP3K13 knockdown significantly increased the malignant phenotypes of HCC cells. Moreover, expression levels of JNK and BAX were significantly reduced after MAP3K13 knockdown in HCCM and Huh-7 cells, while the expression level of Bcl was elevated.

## Discussion

Bioinformatics has now emerged as a viable method for screening potential clinically useful molecular targets [35,36]. In this investigation, it was observed that *MAP3K1, MAP3K3, MAP3K5, MAP3K10*, and *MAP3K15* were upregulated in HCC tumor tissues, whereas *MAP3K7, MAP3K8, MAP3K9*, and *MAP3K11* were downregulated in tumor tissues in GSE14520. Although neither prognostic value nor diagnostic value was observed in *MAP3K7* in our assessments, studies have indicated that *MAP3K7* was a chock block in tumorigenesis of HCC [37–39]. Beyond that, although we found no significantly different levels of expression of *MAP3K6* between the tumor and the normal tissues, we only could identify a single study which suggested that *MAP3K6* may be involved in the dynamics underlying self-renewal and differentiation of tumor progenitor cells [40].

In our diagnostics-related analyses, we identified that *MAP3K9* (also known as *MLK1*) demonstrated good diagnostic efficacy in HBV-related HCC for the first time, and we believe that this feature of *MAP3K* has never been reported before in examinations of HCC. We performed this analysis at the levels of mRNA, in hope that these results could be extended to clinical applications. However, there are still further studies that can be carried out in the future to explore whether the content of the *MAP3K9* gene products taken from samples such as blood and bile from HCC patients could differ those taken from the normal population, as data for these media were not available in the present study. As MAP3K9 has been reported to activate the c-Jun N-terminal kinase (JNK) MAPK signal pathway, consequently, it can act to help regulate two pivotal downstream targets in MAPK-based cascades, including p38 and extracellular signal-regulated kinase (ERK) [41]. The c-Jun N-terminal kinase (JNK) MAPK pathway is known to be mainly involved in the dynamics underlying cell survival and programmed cell death, and it can influence migration and mesenchymal-epithelial transitions (EMT) as well [42–45]. The function and regulation of the MAPK signaling pathway in HCC patients have been extensively studied, but whether *MAP3K9* plays a key role in liver cancer, as well as its mechanisms, remained unknown. Thus, our study could provide information leading to the development of a novel target or biomarker for further application in the study of MAPK signaling pathways and their dynamics, which could influence HCC.

*MAP3K13*, also known as leucine zipper-bearing kinase (LZK), is a member of the mixed lineage kinase (MLK) family, a subfamily of *MAP3Ks* [46]. The survival analyses we used in our study revealed that upregulation of *MAP3K13* was associated with good OS outcomes in HBV-related HCC patients. Prior to our research, MAP3K13 has never been observed to be correlated with survival of HCC patients via the MAP3K13-TRIM25-FBXW7α-Myc axis [47]. A similar type of regulatory pattern has been reported in breast cancer in which microRNA-206 impacted cancer cell proliferation by inducing restraint of *MAP3K13* [48]. It has also been reported that *MAP3K13* may play key roles in the dynamics underlying tumors via impacting the NF-kappaB (NF-ĸB) [49], JNK [50], and the p53 [48,51] signaling pathways. It is also worth noting that the JNK cascade in which MAP3K13 is an indispensable component, plays an important role in stress-related response and in apoptosis that can be induced by various stimuli [52]. In this study, we observed that there was a lower level of expression of *MAP3K13* in cancerous tissues. Hence, we considered that the carcinogenesis of HBV-related HCC samples might have partly resulted from insufficient apoptosis caused by decreased levels of expression of MAP3K13.

In our investigations, *MAP3K15* was also identified to be associated with OS rates of HBV-related HCC. The physiological impacts of *MAPK3K15* are poorly understood. *MAPK3K15* is also known as apoptosis signal-regulating kinase 1(ASK3) and was named in recent years based on sequence homology of ASK1 and ASK2. Previous research has indicated that *MAPK3K15* was required for cell death and its production can be induced by anti-Fas monoclonal antibodies, tumor necrosis factor alpha (TNF), or oxidative stress [53]. Thus, our findings might have suggested that the *MAPK3K15* gene could be included as a new and crucial member in apoptotic signaling kinase and could play a key role in signal transduction pathways related to apoptotic cell death triggered by cell stress. Furthermore, our results suggested that *MAP3K15* might also play a role in the dynamics and mechanisms underlying HCC through its corresponding roles aforementioned in relation to apoptosis.

In addition to findings from survival analyses for each single *MAP3K* gene to help predict the prognoses for HBV-related HCC patients, we have identified possible prognostic targets and conducted the bioinformatics analysis of these targets. We have also integrated genes with significant prognostic value into a prognostic signature model. The prognostic signature helped to delineate data based upon differences and cutoffs between high- and low-risk groups based upon high and low levels of expression of *MAP3K13*/*MAP3K15*. In the nomogram, we were able to intuitively observe measures of the influence of each variable on prognosis upon certain values. BCLC was the most important prognostic variable in our assessment, which indirectly reflected the rationality and practicability of BCLC in liver cancer staging. Although not as effective as BCLC, we did find that *MAP3K13* and *MAP3K15* were better than AFP and tumor size in predicting prognosis. Validation plots of the nomogram for 1-, 3-, and 5- year OS rates showed a good match of predicted and observed data.

Results of GSEA revealed that low levels of expression of *MAP3K13* and *MAP3K15* were involved in dynamics underlying cancer development, and these were essentially consistent with the results of our own study. *MAP3K13* was found to be commonly expressed, but its expression was at a low level in cancer tissues, and HCC patients with a low level of expression of *MAP3K13* typically have bad prognosis, which suggested that *MAP3K13* was a tumor suppressor gene. Similarly, the result might be the same for *MAP3K15*.

There are some defects in our study, which could be improved at the next stage of this work. First, the sample size was not very large. The sample was also derived from a single center, which could only be or might mainly be representative of the characteristics of the specific site of that center. Second, we failed to conduct further experiments to verify the potential mechanisms identified in this study as this was a large endeavor. We expect that we will gradually make improvements in follow-up work.

Although there were several limitations in our investigation, we have helped to uncover the correlation between *MAP3Ks* and the prognosis/diagnosis of HBV-related HCC patients. In conclusion, we observed and determined the diagnostic value of *MAP3K9* in HBV-related HCC and the prognostic value of *MAP3K13* and *MAP3K15* in HBV-related HCC in this study. We established a nomogram based upon clinical characteristics and levels of expression of genes to help us accurately calculate the risk score of each patient. The prognostic signature based upon the levels of expression of *MAP3K13* and *MAP3K15* was satisfactory, and it predicted the survival fit with the actual one in a high degree.

## Conclusion

This article systematically studied the clinical value of MAP3K family members in HCC. We found that *MAP3K9* might be a potential clinical diagnostic indicator in HCC with satisfactory diagnostic efficacy. *MAP3K13* and *MAP3K15* might be useful in predicting the prognosis of HCC patients. Subsequent cytological tests demonstrated that MAP3K13 induced apoptosis of HCC cells by activating the JNK pathway. Knockdown of MAP3K13 significantly enhanced cell proliferation and affected the sensitivity of HCC cells to apoptosis-inducing agents. Our study showed that *MAP3K13* is a tumor suppressor and the genetic regulation of *MAP3K* significantly affects the JNK signaling pathway and apoptotic phenotype of HCC cells.

## Supplementary Material

Supplemental Material

## Data Availability

The data sets used and/or analyzed during the current study are available from the corresponding author on reasonable request.
