# Supplementary material for: Clinical significance of mitogen-activated protein kinase kinase kinases in hepatitis B virus -related hepatocellular carcinoma and underlying mechanism exploration
Source: Bioengineered. 2022 Mar 21;13(3):6818–37. doi: 10.1080/21655979.2022.2037224 (PMC9278978; doi:10.1080/21655979.2022.2037224)
Supplement: Supplemental Material [file KBIE_A_2037224_SM1308.docx]

Table S1. Basic characteristics of 212 patients with hepatocellular carcinoma.

| Variables | Patients | Recurrence-free survival | | | |  | Overall survival | | | |
| --- | --- | --- | --- | --- | --- | --- | --- | --- | --- | --- |
|  | (n=212） | No. of events | MRT  (months) | HR (95% CI) | P |  | No. of events | MST (months) | HR (95% CI) | P |
| Age(years) |  |  |  |  |  |  |  |  |  |  |
| ≤60 | 175 | 96 | 45.9 | ref. |  |  | 69 | NA | ref. |  |
| >60 | 37 | 20 | 48 | 0.974(0.602-1.578) | 0.916 |  | 13 | NA | 0.8643(0.478-1.564) | 0.63 |
| Missing | 0 |  |  |  |  |  |  |  |  |  |
| Gender |  |  |  |  |  |  |  |  |  |  |
| Female | 29 | 10 | NA | ref. |  |  | 8 | NA | ref. |  |
| Male | 183 | 106 | 40.1 | 2.143(1.120-4.100) | 0.021 |  | 74 | NA | 1.704(0.821-3.534） | 0.152 |
| Missing | 0 |  |  |  |  |  |  |  |  |  |
| Multinodular |  |  |  |  |  |  |  |  |  |  |
| Single | 167 | 90 | 49.1 | ref. |  |  | 59 | NA | ref. |  |
| Multiple | 45 | 26 | 28.7 | 1.216(0.785-1.883) | 0.382 |  | 23 | 47.9 | 1.607(0.992-2.604) | 0.054 |
| Missing | 0 |  |  |  |  |  |  |  |  |  |
| Tumor size |  |  |  |  |  |  |  |  |  |  |
| ≤5 cm | 137 | 73 | 51.1 | ref. |  |  | 46 | NA | ref. |  |
| >5 cm | 74 | 43 | 28.4 | 1.409(0.966-2.056) | 0.075 |  | 36 | 53.3 | 1.975(1.274-3.060) | 0.002 |
| Missing | 1 |  |  |  |  |  |  |  |  |  |
| Cirrhosis |  |  |  |  |  |  |  |  |  |  |
| NO | 17 | 5 | NA | ref. |  |  | 2 | NA | ref. |  |
| YES | 195 | 111 | 37.9 | 2.612(1.066-6.402) | 0.036 |  | 80 | NA | 4.335(1.065-17.638) | 0.041 |
| Missing | 0 |  |  |  |  |  |  |  |  |  |
| BCLC |  |  |  |  |  |  |  |  |  |  |
| 0 | 20 | 6 | NA | ref. |  |  | 2 | NA | ref. | 0 |
| A | 143 | 74 | 51.6 | 2.050(2.892-4.711) | 0.091 |  | 48 | NA | 4.119(1.001-16.951) | 0.05 |
| B | 22 | 15 | 26.9 | 4.019(1.550-10.421) | 0.004 |  | 12 | 46.1 | 8.992(2.005-40.320) | 0.004 |
| C | 27 | 21 | 8.9 | 6.163(2.477-15.333) | <0.001 |  | 20 | 13.6 | 18.993(4.419-81.632 | <0.001 |
| Missing | 0 |  |  |  |  |  |  |  |  |  |
| Serum AFPφ |  |  |  |  |  |  |  |  |  |  |
| ≤300 ng/ml | 115 | 62 | 48 | ref. |  |  | 39 | NA | ref. |  |
| >300 ng/ml | 94 | 54 | 35.2 | 1.200(0.833-1.728) | 0.328 |  | 43 | NA | 1.546(1.002-2.385) | 0.049 |
| Missing | 3 |  |  |  |  |  |  |  |  |  |

Abbreviation: AFP, α-fetoprotein; BCLC, Barcelona Clinic Liver Cancer; CI, confidence interval; HR, hazard ratio; MRT, median recurrence time; MST, median survival time; NA, not available; OS, overall survival; RFS, recurrence-free survival.
